# A Study of The Effect of Demand Uncertainty for Low-Carbon Products Using a Newsvendor Model

**DOI:** 10.3390/ijerph14111276

**Published:** 2017-10-25

**Authors:** Shaojian Qu, Yongyi Zhou

**Affiliations:** Business School, University of Shanghai for Science and Technology, Shanghai 200093, China; qushaojian@usst.edu.cn

**Keywords:** low-carbon product, newsvendor model, trust coefficient, consumer aversion coefficient, demand uncertainty

## Abstract

This paper studies the effect of uncertain demand on a low-carbon product by using a newsvendor model. With two different kinds of market scales, we examine a game whereby a manufacturer produces and delivers a single new low-carbon product to a single retailer. The retailer observes the demand information and gives an order before the selling season. We find in the game that if the retailer shares truthful (or in contrast unreal or even does not share) forecast information with the manufacturer, the manufacturer will give a low (high) wholesale price through the sequence of events. In addition, as a policy-maker, the government posts a subsidy by selling the low-carbon product per unit. The manufacturer creates a new contract with a rebate for the retailer. We also take the consumer aversion coefficient and truth coefficient as qualitative variables into our model to study the order, pricing, and expected profit for the members of supply chain. The research shows that uncertain demand causes a the major effect on the new low-carbon product. Thereby, we suggest the retailer should share more truthful information with the manufacturer.

## 1. Introduction

As a result of economic development, our living standards have advanced. Consequently, the environment is currently facing many serious challenges in the context of both developing and developed countries. In China, the total waste water discharge has undergone sustained growth, rising from 5.14 billion tons in 2006 to 7.35 billion tons in 2015. In recent years, many academic studies have shown that the low-carbon consumption, low-carbon energy, carbon emission reduction, and government policy are the major factors for improving environmental quality. For example, Rogers et al. indicate the biomass utilized in the current economy (365 million dry tons as of 2014) is estimated to displace approximately 2.4% of fossil energy consumption and avoid 116 million tons of ${\mathrm{CO}}_{2}$-equivalent emissions, while the 2030 target could displace 9.5%. Thus, a sustainable market should be achieved for a low-carbon economy [1]. Gong et al. investigated the input–output efficiency of economic indicators and energy consumption in Beijing city, and proposed an effective basis for the scientific and reasonable arrangement of Beijing city’s short-term climatic resources and energy-economic development [2]. In order to alleviate global warming, governments and organizations have created laws or regulations to more strictly prohibit the carbon emissions of firms. For example, the Carbon Trust of Britain developed the Carbon Reduction Label Scheme as a pioneer plan for the creation of low-carbon product certification in 2006. In China, the government created some policies and regulations encouraging firms to produce low-carbon products (for example electric cars and green energy) by using financial subsidies. In addition, Chen and Gong integrated environmental regulations as qualitative variables into the energy consumption evaluation system by constructing a data envelopment analysis [3].

Although the government is fully aware of the potential dangers of environmental pollution, the majority of the firms and consumers are unaware of the importance of low-carbon consumption in practice. As a result, the problems of global warming, waste water, and exhaust gas emissions require the government to make some efforts to encourage firms to implement low-carbon production as well as consumers to practice low-carbon consumption.

To address these issues, we study a game whereby a single manufacturer produces a single new low-carbon product which is sold to a single retailer; the retailer observes forecast information before placing an order through a newsvendor model. The basic assumption of the product is uncertain demand on a short-term basis. We know that if the retailer shares more truthful (or in contrast unreal or does not share) forecast information with the manufacturer, he will gain a low (high) wholesale price through the sequence of events. This requires the manufacturer to set a contract, denoted by the trust coefficient, to coordinate the supply chain. As a result, the retailer must share more information, while the manufacturer has greater risks in the game. In this paper, we focus on the following areas: (1) We examine a game whereby a single manufacturer produces and delivers a single low-carbon product to a single retailer, while the retailer observes the demand information and gives an order prior to the selling season; (2) We study the effect of demand uncertainty on the manufacturer’s optimal quantity with the trust coefficient and rebate the incorporating consumer aversion coefficient; and (3) We explore the impact of the low-carbon products in reducing emissions and improving the quality of the environment by using 2006–2015 data of the main pollutant emissions in waste water and gas by region in China.

Surprisingly, even if the demand is active, the retailer is not willing to share truthful forecast information to the manufacturer. In order to encourage the retailer to share more, the manufacturer will set a rebate contract under two kinds of different demands. In addition, we suppose the consumers have an aversion to the product in a new market, and the consumer aversion as a qualitative variable strongly affects the order and expected profits of the members.

This paper also uses 2006–2015 data of the main pollutant emissions in waste water and gas by region in China, and shows that Chinese industrial pollution, living pollution, and other factors contribute to the environmental problem. The survey elaborates that we should improve energy efficiency, use low-carbon products, and reduce carbon emissions.

Our analysis offers a novel rationale for the new low-carbon product under uncertain demand. We establish an effective contract in which the retailer obtains a low wholesale price, while the manufacturer also enlarges the coverage of the new product in the market. This means the members operate in a win–win case. In addition, the analysis of the game suggests that the retailer should share more truthful forecast information, and the manufacturer should set some new mechanisms to drive the green supply chain to become perfectly coordinated. Finally, the government and firms should not only strive to advance the low-carbon industry, but the consumers should strengthen their awareness of low-carbon consumption.

## 2. Related Literature

The central contribution of our work with respect to previous studies is that the same model is framed in both short-term and uncertain demand—the game comprises a single retailer and a single manufacturer in dynamics that may yield a win–win situation.

### 2.1. Newsvendor Model

The newsvendor model has been extensively studied in the supply chain for decades [4,5,6,7]. Song and Zhao analyzed how different buyers with bounded rationality or who are waiting for a discount choose a purchase policy in a newsvendor system. In particular, they believe the newsvendor decides the order and price [8]. However, the main difference between our work and their paper is that we focus on the uncertain demand that strongly affects the manufacturer’s behaviors, which is another contribution to the newsvendor models [9,10,11,12,13,14].

Tsao studies how trade credit is beneficial to coordinating the supply chain. In their paper, they analyze three methods of carbon emissions (carbon caps, carbon caps with trade and carbon tax) by formulating a newsvendor model under trade credit with the risk of default, and the outcome shows the optimal trade period, order, and price are given [14]. In our paper, we take (1) information sharing; (2) the trust coefficient; and (3) the consumer aversion coefficient into their newsvendor models, and therefore we further characterize how the low-carbon product has impacts on the manufacturer’s game.

### 2.2. Uncertain Demand

Many academic studies have shown that uncertain demand is a factor in coordinating the supply chain. For example, for manufacturing, the order penetration point is affected by the production time, demand, and inventory and proposes some rational suggestions [15,16]. In addition, Liu studies how the supply reliability affects the retailer’s performance based on joint marketing and inventory decisions, and their model emphasizes the impact of marketing efforts on consumer demand. In order to advance the supply reliability, importantly, their paper analyzes the investment decisions in new technologies [17]. Similarly, in our paper, we study the effect of uncertain demand on the new low-carbon product. However, we mainly state the game in which the retailer observes demand information and gives an order; the manufacturer posts a wholesale price and produces the product. In the literature, how the uncertain demand advances the supply chain coordination is studied, by considering the inventory, production, and retail price [18,19,20,21,22]. However, our model considers information sharing, the consumer aversion coefficient, and the trust coefficient in the supply chain with uncertain demand.

### 2.3. Information Sharing

In addition, an increasing number of scholars have argued that information sharing has been important to the supply chain coordination for decades [23,24,25]. Some papers propose knowledge management systems best suited to reducing misalignment and improving operational performance in the terms of efficiency and effectiveness, which will support the members to share more truthful information, thereby coordinating the supply chain [26,27,28,29,30,31]. Moreover, trust is vital in information sharing, emphasizing that if makers establish the trust mechanism, consensus can be achieved [32,33,34]. Our model is also involved with the literature that studies the information sharing of the members in the supply chain. The sequence of events shown by Cachon and Lariviere showed that the manufacturer shares information with the supplier, then the supplier decides his capacity, and the manufacturer provides a final order in the overall selling season [35]. In contrast to their model, we find that the supply chain has a special stage at which the manufacturer sets a new mechanism to entice the retailer to share more truthful forecast information before giving an order.

In a recent working paper, Choi examined the inventory decisions of fashion retailing by implementing the quick response system. In his paper, he studied the cases under which fashion retailing is short term, facing uncertain demand, and he finds that the fashion retailers have access to the demand information by advancing in technology [36]. However, we find that the retailer does not share forecast information or shares unreal information with the manufacturer when his expected profit is not operated optimally. A key difference is that our paper assumes the market plays in two states: active and depressed, which leads the members to choose different order and pricing policies. The conclusion is that there can be more or less information shared depending on the different scales.

### 2.4. Low-Carbon Product

Our research is also related to the emerging field of low-carbon [35,37,38]. Rao et al. propose a new evaluation system for supplier selection from a sustainability perspective by integrating economic, environmental and social aspects [39]. In particular, a linguistic two-tuple VIKOR method(a multi-criteria decision making or multi-criteria decision analysis method) [40,41] and a new two-stage compound mechanism [42] study low carbon supplier selection under the development mode of low carbon economy. Fera elaborates the effect of the electricity technology for renewable energy and the quality of the environment [43,44]. In addition, Zhu analyzed the effect of consumer preference on low-carbon products by adopting a novel emission-sensitive demand function. In contrast to our paper, this paper supposes that the consumers are willing to choose the low-carbon product, and it was found that if there is a lack of internal moral self-discipline as well as external regulation, there will be less eco-friendly production than in traditional production [45]. In our paper, we take the consumer aversion as a qualitative variable in our newsvendor model. In addition, Liu showed that their model solves the low-carbon supply chain resource allocation problem by designing a novel quantum chaos neural network algorithm [38]. However, in our model, we study the optimal order quantity and pricing of the low-carbon product with a game between a single manufacturer and a single retailer.

### 2.5. Government Policy

Furthermore, our paper is also involved in the literature that studies government policy for low-carbon products and carbon emission reductions (which began with an influential paper by Seifritz [46]), such as policies to improve the quality of the environment [47,48,49,50,51]. These studies focus on carbon emissions with respect to morals, policies, laws, etc. However, our model studies how the government entices the green supply chain to operate optimally by setting subsidies for low-carbon products.

The rest of the paper is organized as follows. In Section 2 we present a model to describe the problem of the supply chain game. In Section 3, we analyze the impact of the consumer aversion on the manufacturer’s expected profit. Section 4 compares the trust coefficient and rebate, and we characterize how the members create policy in the green supply chain. Section 5 shows the numerical application to illustrate the efficiency of our methods. Section 6 concludes the paper.

## 3. The Model

### 3.1. A Newsvendor Model

The newsvendor model is a mathematical model in operations research, and applied economics are used to determine optimal order quantity under uncertain demand for a product. In our paper, the retailer’s order policy can be described by the newsvendor model [52,53].

### 3.2. Players

Considering that a two-level supply chain consists of a single manufacturer and a single retailer for a low-carbon product that will last for one new market, we examine a game in which the retailer observes the demand information and sends out an order; the manufacturer produces and delivers the product before the selling season.

### 3.3. Notations

We use “$q_{r}$” to denote the retailer’s order quantity, and “$q_{m}$” to denote the manufacturer’s production quantity. The other notations to be used are defined in Table 1.

### 3.4. The Game

In this paper, we examine a game where the retailer observes the demand of the new low-carbon product and gains the information at the beginning of selling season, while the manufacturer posts a wholesale price and produces the product after receiving the order. In order to operate the expected profit optimally, we find that the new mechanism is set by the manufacturer to entice the retailer to share more truthful information especially when the demand is stochastic. Moreover, the retailer shares truthful or unreal (or does not share) information with the manufacturer in the full selling season, and thereby the manufacturer will give a low or high wholesale price before receiving the order.

### 3.5. The Subsidy

The government as a policy-maker wishes to promote the utilization in the low-carbon industry and stimulate sustainable development within it. Given that low-carbon consumption reduces the emissions, we suppose that the retailer (the manufacturer) will gain the government’s subsidy $L_{1}$ ($L_{2}$), if he orders (produces) the low-carbon product per unit in the full selling season.

Since the manufacturer produces a single short-term low-carbon product at the beginning of selling season, and the retailer’s order policy can be described by the newsvendor model, we can get the retailer’s profit:

(1) $$\begin{array}{cc} \pi_{r}\left(q\right) & =pMin\left(q,D\right)+sMax\left(0,q-D\right)-wq+L_{1}q-c_{r}\\ & =pMin\left(q,D\right)+sMax\left(0,q-D\right)+\left(L_{1}-w\right)q-c_{r}, \end{array}$$

where $c_{r}$ represents the other costs of the *R*, such as the cost of advertising, sales costs, and transportation costs. Then, we have the expected profit:

(2) $$E\left[\pi_{r}\left(q\right)\right]=\left(p-w+L_{1}\right)q+\left(s-p\right)\int_{0}^{q}F\left(x\right)dx-c_{r}.$$

Maximizing his expected profit in Equation (2), we can get

(3) $$q_{r}=F^{-1}\left(\frac{p-w+L_{1}}{p-s}\right).$$

Next, we analyze the whole supply chain’s expected profit using

(4) $$E\left[\pi_{s}\left(q\right)\right]=\left(p-c+L_{1}+L_{2}\right)q+\left(s-p\right)\int_{0}^{q}F\left(x\right)dx-c_{s},$$

where $c_{s}$ represents the other costs in the whole supply chain. Then, maximizing the $E[\pi_{s}\left(q\right)]$, we have

(5) $$q_{s}=F^{-1}\left(\frac{p-c+L_{1}+L_{2}}{p-s}\right).$$

It is clear that $q_{r}<q_{s}$. This means that the overall supply chain’s optimal quantity is larger than the retailer’s optimal order quantity. Hence, the supply chain is uncoordinated.

As a new low-carbon product, the manufacturer wants to expand the coverage of the product in the market, which leads the retailer to order more products at the beginning of the selling season. The following proposition shows that the retailer will accept the manufacturer’s strategy.

**Proposition** **1.** As a rational policy-maker the retailer will accept the manufacturer’s strategy (i.e., the manufacturer wishes his retailer to order more products before the selling season, which will enlarge the coverage of the low-carbon product in the market) if and only if demand is active and $w\in (c,s+L_{1})$.

All proofs can be found in Appendix A.

From Proposition 1, we find that the manufacturer’s wholesale price is an important variable in their decisions in the supply chain, especially when the demand is large enough. On the one hand, we believe that when the manufacturer gives a low wholesale price, his retailer will post a low retail price, and thus the retailer’s product will attract more consumers by ordering more. On the other hand, for a new low-carbon product, expanding the coverage of the product in the market is particularly vital, as it will promote the development of low-carbon economy, thereby improving the quality of the environment.

### 3.6. The Sequence of Events

In this section, we first assume that the retailer and manufacturer are cooperative and other factors are not involved in the green supply chain. The retailer shares truthful information with the manufacturer at t=0. At t=1, the manufacturer gives the wholesale price after receiving the demand information. Then, the retailer places an order at t=2, and at t=3, the manufacturer produces and delivers the order.

In addition, we have the manufacturer’s expected profit:

(6) $$E\left[\pi_{m}\left(q\right)\right]=\left(w-c+L_{2}\right)q-c_{m}.$$

Intuitively, the manufacturer’s optimal supply quantity $q_{m}$ is the largest allowable lot size in Equation (6).

**Figure 1:**
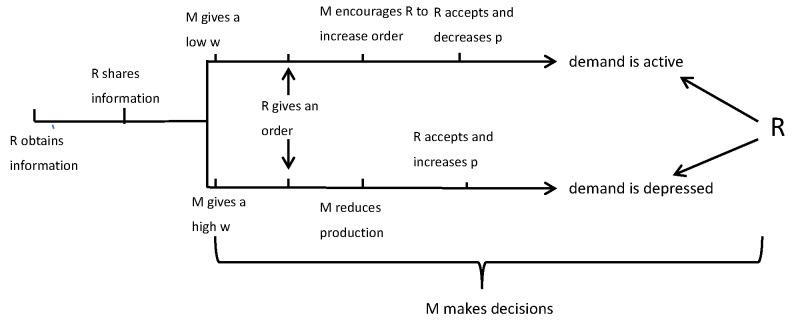
The cooperative case.

Hence, we find that when the demand is great, the manufacturer will encourage the retailer to increase his order quantity after posting a wholesale price in Figure 1 (We use “*R*” to denote the retailer, and “*M*” to denote the manufacturer). At the same time, from Proposition 1 we know if $w\in (c,s+L_{1})$, the retailer will accept the manufacturer’s strategy. However, when the demand is depressed, the manufacturer will give a high wholesale price, instead of expanding the production level. In this case, the retailer will have a lower order quantity and a higher price. We find that when $w\in (c,s+L_{1})$, the retailer will still accept the manufacturer’s decision, since his expected profit can operate optimally. The analysis of the cooperative case of the retailer and manufacturer shows if the retailer shares truthful information with the manufacturer, they may be yielding a win–win situation by making a rational policy. As a result, the supply chain is coordinated.

**Figure 2:**
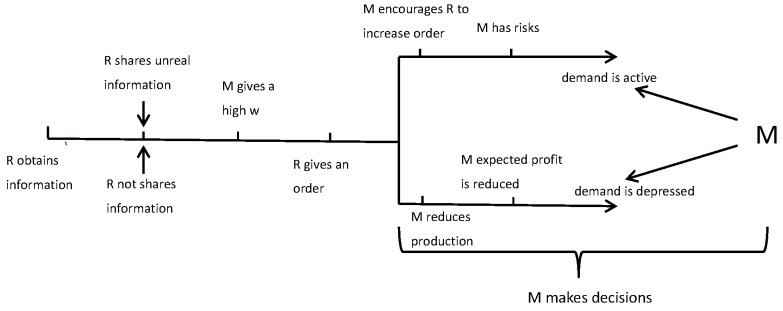
The non-cooperative case.

Next, we suppose that the retailer and manufacturer are non-cooperative and other factors are not involved in the green supply chain. At t=0, the retailer observes the demand information, and then he shares unreal demand information or even does not share information to the manufacturer at t=1. At t=2, the retailer sends out an order, and then the manufacturer decides the wholesale price. The supply chain has a special period at t=3. We can see that the supply chain is uncoordinated in Figure 2.

## 4. The Analysis of the Decisions of the Manufacturer

In this section, we analyze the decisions that have an impact on the expected profit. Our model now tries to solve the following questions. Firstly, when we consider other factors such as consumer aversion, trust, and the rebate, how will the members coordinate the supply chain? Secondly, for new mechanisms, how will the members achieve a win–win situation in the green supply chain?

### 4.1. Consumer Aversion Coefficient

As a new low-carbon product, the demand is uncertain. Zhang considered how the default risk influences the manufacturer’s expected profit [11]. However, we suppose that customers have a aversion to the product, denoted by γ (0<γ<1), and when the sum of retailer’s income, subsidy, and the salvage value of the left stock are larger than the quantity that should be paid by the retailer, customers will have perfect preference, otherwise the customers will have a aversion coefficient. Hence, we can obtain the manufacturer’s profit:

(7) $$\pi_{m}\left(\gamma ,q\right)=\left\{\begin{array}{cc} \left(w+L_{2}-c\right)q-c_{m}, & \mathrm{if}\;D\geq \frac{(w-s)q}{p+L_{1}-s}\\ \gamma \left[\left(p+L_{1}\right)D+s\left(q-D\right)\right]+\left(1-\gamma \right)wq-cq+L_{2}q-c_{m}, & \mathrm{otherwise} \end{array}\right.$$

Here, we say the retailer gets the government’s $L_{1}$ when he sells a low-carbon product per unit, rather than through orders. The condition $D\geq \left(w-s\right)q/\left(p+L_{1}-s\right)$ is equivalent to $\left(p+L_{1}\right)D+\left(q-D\right)s\geq wq$, which means that the sum of the values from the subsidy, left stock, and sold products is larger than the quantity that should be paid by the retailer. We have just analyzed that how the manufacturer’s optimal supply quantity influences the supply chain coordination, and we find that his $q_{m}$ is the largest allowance lot size from Proposition 1. This result is consistent with the previous studies where the consumer aversion coefficient is neglected. However, the following Theorem shows that our model finds a new conclusion for the low-carbon product.

**Theorem** **1.** For the low-carbon product, when the consumer aversion coefficient is involved, the manufacturer has a unique maximizer of $E[\pi_{m}\left(\gamma ,q\right)]$.

Comparing Equations (6) and (7), when the consumer aversion coefficient is considered, the manufacturer’s expected profit does not increase in $q_{m}$, but we can yield a unique optimal supply quantity $q_{m}$ to maximize the expected profit using Theorem 1. As a new low-carbon product, the consumer aversion coefficient is a significant factor in how to enlarge the marketing rate and promote the popularity.

Generally speaking, if the retailer’s order $q_{r}$ is larger than the manufacturer’s optimal lot size $q_{m}$, we find that the manufacturer will deliver $q_{m}^{\prime }\left(q_{m}\leq q_{m}^{\prime }\leq q_{r}\right)$ to the retailer to optimize his expected profit and reduce risks. While the $q_{r}$ is smaller than $q_{m}$, we find that the manufacturer will post a low *w* to encourage the retailer to share more information and order more than the $q_{r}$, which leads the manufacturer to take more risks. As a result, the manufacturer sets a new mechanism in the overall selling season.

Wu studies trust based on estimation and aggregation methods as part of a visual consensus model for multiple criteria group decision-making with incomplete linguistic information [33]. However, our paper integrates trust as qualitative variables into the supply chain to study using newsvendor how the optimal supply quantity, pricing, and information sharing are affected.

### 4.2. Trust Coefficient

When the product demand is stochastic and the manufacturer’s information is from the retailer, we suppose that the manufacturer sets a mechanism, denoted by λ and λ∈[0,1], to represent the retailer’s information valuation. In this paper, larger λ means a lower *w*. Therefore, the retailer’s expected profit is constructed as:

(8) $$E\left[\pi_{r}\left(\lambda ,q\right)\right]=\left(p-\frac{1}{\lambda }w+L_{1}\right)q+\left(s-p-L_{1}\right)\int_{0}^{q}F\left(x\right)dx-c_{r}.$$

We know that the retailer wishes (other things being equal) to share more truthful information to his manufacturer, so he then will obtain a low wholesale price in Equation (8). The following Theorem shows the trust coefficient is a key variable for the retailer’s order quantity.

**Theorem** **2.** We can find that the retailer’s optimal order quantity is increasing with the trust coefficient, and if the product’s demand is great, the retailer prefers to share more truthful information with the manufacturer.

Theorem 2 emphasizes the effect of trust for information sharing with each other in the supply chain. We find that the manufacturer establishes the truth mechanism in the game, which will entice the retailer to share more truthful information if the demand is great. Moreover, the retailer prefers to share more to obtain a low wholesale price at the beginning of the selling season. As a result, the manufacturer sets the trust coefficient as reasonable and intelligent in the game.

We next analyze the manufacturer’s expected profit considering the trust coefficient. First and foremost, let $B=\frac{(\frac{1}{\lambda }w-s)q}{p+L_{1}-s}$, and we have his profit:

(9) $$\pi_{m}\left(\lambda ,\gamma ,q\right)=\left\{\begin{array}{cc} \left(\frac{1}{\lambda }w+L_{2}-c\right)q-c_{m}, & \mathrm{if}\;D\geq \frac{(\frac{1}{\lambda }w-s)q}{p+L_{1}-s}\\ \gamma \left[\left(p+L_{1}\right)D+s\left(q-D\right)\right]+\left(1-\gamma \right)\frac{1}{\lambda }wq-cq+L_{2}q-c_{m}, & \mathrm{otherwise} \end{array}\right.$$

Combined with the the consumer aversion coefficient and trust coefficient, the following Theorem shows how the manufacturer’s optimal lot size operates optimally in the green supply chain.

**Theorem** **3.** *We have the following two new conclusions (let $H=\frac{\frac{1}{\lambda }w+L_{2}-c}{\gamma (\frac{1}{\lambda }w-s)}$):*

*1.*  For any $\gamma \in [\frac{\left(L_{2}-c+s\right)f\left(F^{-1}\left(H\right)\right)}{F^{-1}\left(H\right)s},1]$, if $\frac{\gamma wF^{-1}\left(H\right)}{\gamma F^{-1}\left(H\right)s-\left(L_{2}-c+s\right)f\left(F^{-1}\left(H\right)\right)}<\lambda \leq 1$, the manufacturer’s $q_{m}$ decreases with λ;
*2.*  For any λ∈[0,1], however, the manufacturer’s $q_{m}$ decreases with γ.

For the manufacturer, we know that when both the trust coefficient and consumer aversion coefficient are involved, he will make decisions by combining with the trust coefficient and consumer aversion coefficient from Theorem 3 . On the one hand, from Theorem 2, we learn that a high trust coefficient entices to the retailer to share more. However, when $\frac{\gamma wF^{-1}\left(H\right)}{\gamma F^{-1}\left(H\right)s-\left(L_{2}-c+s\right)f\left(F^{-1}\left(H\right)\right)}<\lambda \leq 1$, the manufacturer’s optimal supply quantity $q_{m}$ decreases with λ, which will be not conducive to enlarging the coverage of the product in the market. On the other hand, for a new low-carbon product, consumer aversion is also directly related to the marketing rate. We further study how the manufacturer and the retailer reduce the consumer aversion coefficient by their efforts.

Hence, the Theorem echoes that the manufacturer wishes the consumer aversion coefficient to be sufficiently small and the trust coefficient sufficiently high.

## 5. Comparing the Trust Coefficient and Rebate

In the above sections, we find that if the demand is great, the manufacturer will encourage the retailer to increase his order quantity. In this section, we characterize how the rebate affects the manufacturer’s decisions.

### 5.1. The Rebate

When the low-carbon product’s demand is large enough, the manufacturer will give a rebate per unit after delivering $q_{m}$ to the retailer in practice, denoted by *b*. Since the demand is large, the retailer will order more to meet the demands of the vast majority of consumers. We believe that as a rationale manufacturer will set a not large rebate *b* as a reward to his retailer, and b∈(0,s). In the general case, the retailer makes a large order to obtain the manufacturer’s rebate. We suppose that the truth coefficient is neglected by the manufacturer, and the retailer expects the product to meet the demands of the majority of customers. Obviously, the demand shows customers’ aversion coefficient γ→0. We then have the manufacturer’s expected profit:

(10) $$E\left[\pi_{m}\left(\lambda ,b,q\right)\right]=\left(\frac{1}{\lambda }w+L_{2}-c-b\right)q-c_{m}.$$

We can see that the manufacturer’s $q_{m}$ is large enough in Equation (10), and he will entice the retailer to order more products. With a rebate, we find that if b→s, the retailer will accept the manufacturer’s strategy decision. However, if b→0, the retailer will have a special stage in which the retailer can further obtain and update demand information.

In particular, when the demand is small, the consumer aversion coefficient is involved. Here, in order to stimulate the retailer’s demand, we assume the manufacturer also gives a rebate $b_{1}\left(b_{1}\in \left(0,s\right)\right)$ after selling the product per unit in the green supply chain. Hence, the manufacturer’s profit is

(11) $$\pi_{m}\left(\lambda ,\gamma ,b_{1},q\right)=\left\{\begin{array}{cc} \left(\frac{1}{\lambda }w+L_{2}-c-b_{1}\right)q-c_{m}, & \mathrm{if}\;D\geq \frac{(\frac{1}{\lambda }w-s)q}{p+L_{1}+b_{1}-s}\\ \gamma \left[\left(p+L_{1}+b_{1}\right)D+s\left(q-D\right)\right]+\left(1-\gamma \right)\frac{1}{\lambda }wq-cq+L_{2}q-c_{m}, & \mathrm{otherwise} \end{array}\right.$$

we let $C=\frac{(\frac{1}{\lambda }w-s)q}{p+L_{1}+b_{1}-s}$ and obtain the expected profit as

(12) $$\begin{array}{cc} E[\pi_{m}\left(\lambda ,\gamma ,b_{1},q\right)] & =\int_{0}^{C}\{\gamma \left[\left(p+L_{1}+b_{1}\right)+s\left(q-D\right)\right]+\left(1-\gamma \right)\left(\frac{1}{\lambda }wq-cq+L_{2}q-c_{m}\right\}f\left(x\right)dx\\ & +\int_{C}^{\infty }\left[\left(\frac{1}{\lambda }w+L_{2}-c-b_{1}\right)q-c_{m}\right]f\left(x\right)dx. \end{array}$$

The simplified form of Equation (12) is

(13) $$E\left[\pi_{m}\left(\lambda ,\gamma ,b_{1},q\right)\right]=\left(\frac{1}{\lambda }w+L_{2}-c-b_{1}\right)q-\int_{0}^{C}\left(p+L_{1}+b_{1}-s\right)\gamma F\left(x\right)dx-c_{m}.$$

In the above section, we know the λ strongly affects the manufacturer’s $q_{m}$. The following Proposition shows the demand has impact on the λ.

**Proposition** **2.** *The following two scenarios hold:*

*1.*  When the demand is large enough and γ→0, we find, however, the retailer may obtain a small λ;
*2.*  When the demand is depressed and γ>0, we find, surprisingly, the retailer may gain a high λ.

For the new low-carbon product, not only does the government give a subsidy, but the manufacturer sets a rebate to the retailer, which will stimulate the retailer to order more. In contrast to Theorem 3, we find that the retailer may not operate his expected profit optimally by setting a rebate under great demand in Proposition 2. In addition, from Theorem 3, we also know that no matter whether the demand is active (γ→0) or depressed (γ>0), in order to enlarge the product’s marketing rate, the manufacturer will make new contracts to coordinate the green supply chain.

## 6. Numerical Study

In the section, we employ a numerical study to further investigate the value of the newsvendor model in the previous sections. Moreover, we use 2006–2015 data of main pollutant emissions in waste water and gas by region in China, and show the Chinese environment is facing serious challenges currently. To provide a better conclusion directly, we try to perform a numerical study to respond to the following questions. Firstly, when the retailer shares truthful or unreal (or does not share) forecast information to the manufacturer, how does the retailer’s expected profit operate optimally? Secondly, when the consumer aversion coefficient is involved, what are the manufacturer’s decisions on setting different mechanisms to coordinate the supply chain ? Thirdly, comparing the trust coefficient and rebate, how do the members of the supply chain achieve a win–win case? Fourthly, why does the government create environment laws and regulations to more strictly prohibit the firms’ carbon emissions and encourage production of low-carbon products?

**Figure 3:**
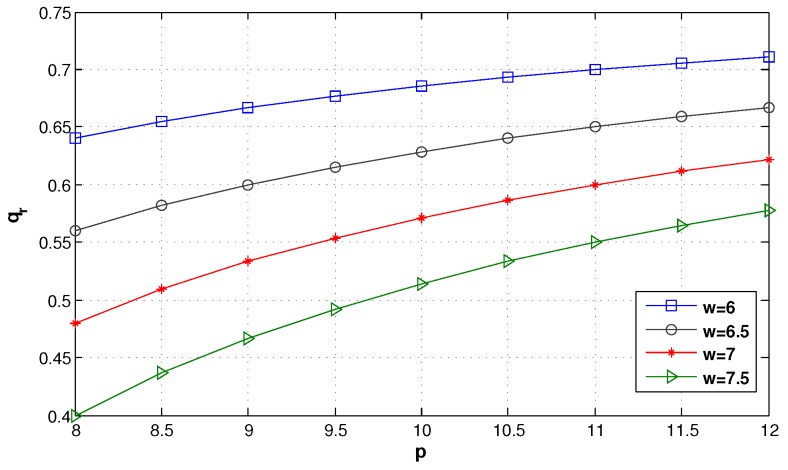
The optimal order quantity of the retailer under different wholesale prices.

**Figure 4:**
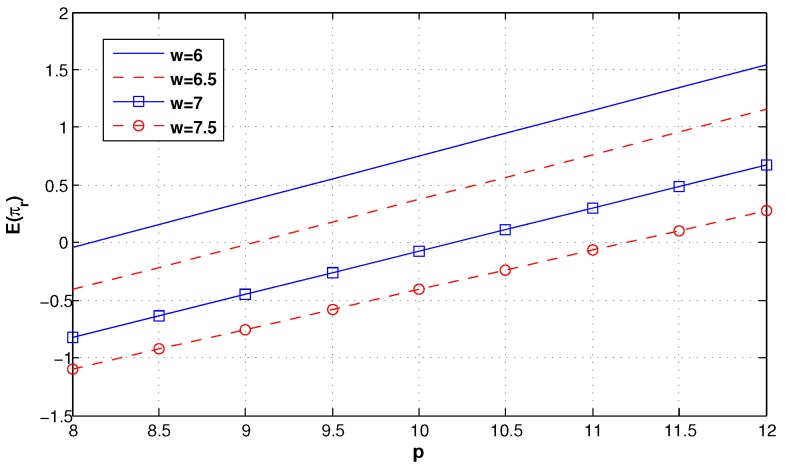
The expected profit of the retailer under different wholesale prices.

We first suppose that other factors are not considered in the green supply chain. Figure 3 shows the impact of the different wholesale prices on the retailer’s optimal order quantity. It can be seen that $q_{r}$ decreases with *w*, and when *w* is low enough, $q_{r}$ weakly increases with *p*. In addition, in Figure 4, we can find that the retailer’s expected profit also decreases with *w*, and when *w* is low enough, however, $E(\pi_{r})$ strongly increases with *p*. Hence, the retailer should share more truthful information to obtain a low wholesale price from the manufacturer.

**Figure 5:**
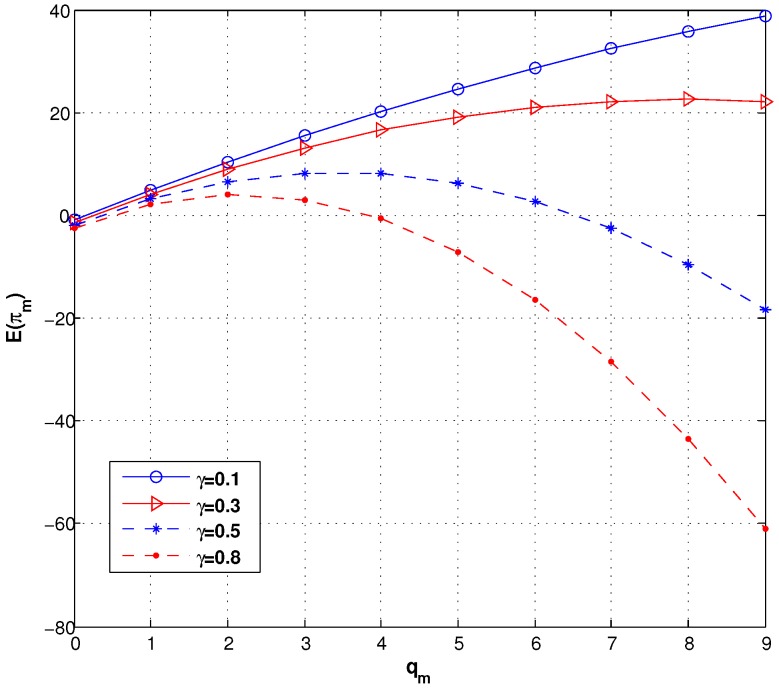
The expected profit of the manufacturer under different γ.

We know the manufacturer’s expected profit increases with $q_{m}$ in Equation (6), so he expects the retailer to increase his order quantity. However, when the trust coefficient and the consumer aversion coefficient are involved, we find that the $E(\pi_{m})$ is concave in $q_{m}$ over the interval $\left[\underline{q_{m}},\bar{q_{m}}\right]$, and therefore there is a maximizer of $E(\pi_{m})$. Figure 5 presents the manufacturer’s expected profit under different consumer aversion coefficients. We know only when γ→0, $E[\pi_{m}\left(\gamma ,q\right)]$ increases with $q_{m}$ in $q_{m}\in \left(0,+\infty \right)$. Hence, it is necessary that the manufacturer set λ as a new mechanism for the retailer.

**Figure 6:**
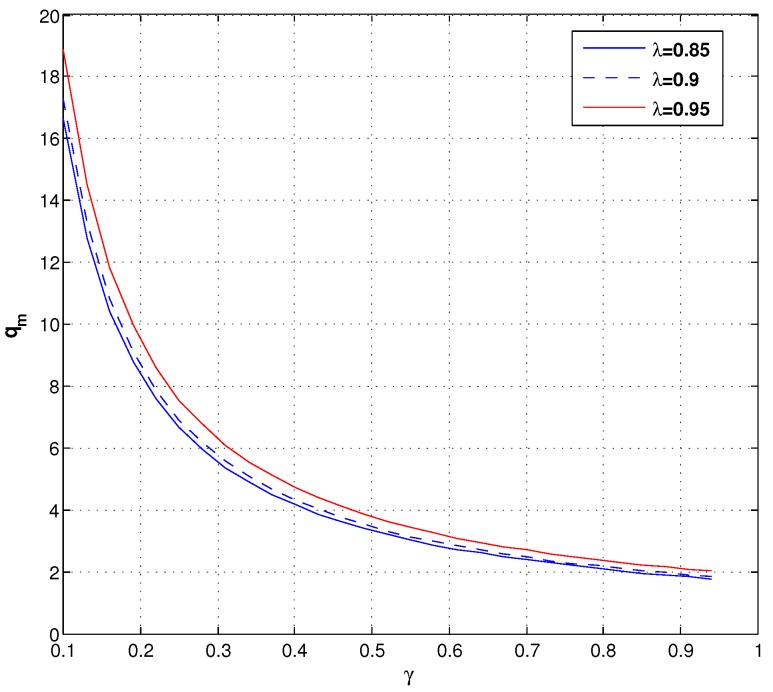
The optimal lot size of the manufacturer under different λ.

Figure 6 demonstrates the manufacturer’s optimal lot size under different values of γ and λ. We can learn that $q_{m}$ decreases with γ under different λ. Descriptions of Figure 4, Figure 5 and Figure 6 mean the manufacturer needs to obtain more forecast information from the retailer to learn the γ.

**Figure 7:**
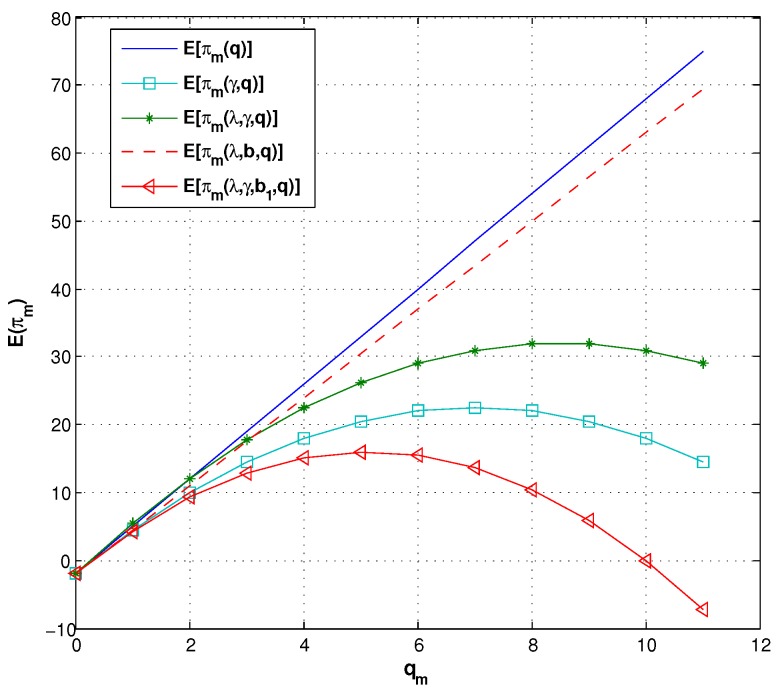
The expected profit of the manufacturer under different contracts.

In particular, Figure 7 compares the manufacturer’s expected profit in different contracts with and without the consumer aversion coefficient. We find the fact that when γ is not considered, $E(\pi_{m})$ increases with $q_{m}$. However, when γ is involved, there is a maximizer of $E(\pi_{m})$, which coincides with Figure 5. We can further learn that the manufacturer’s optimal lot size and expected profit are less, although the manufacturer creates a new contract by setting a rebate.

### 6.1. Social Welfare

**Figure 8:**
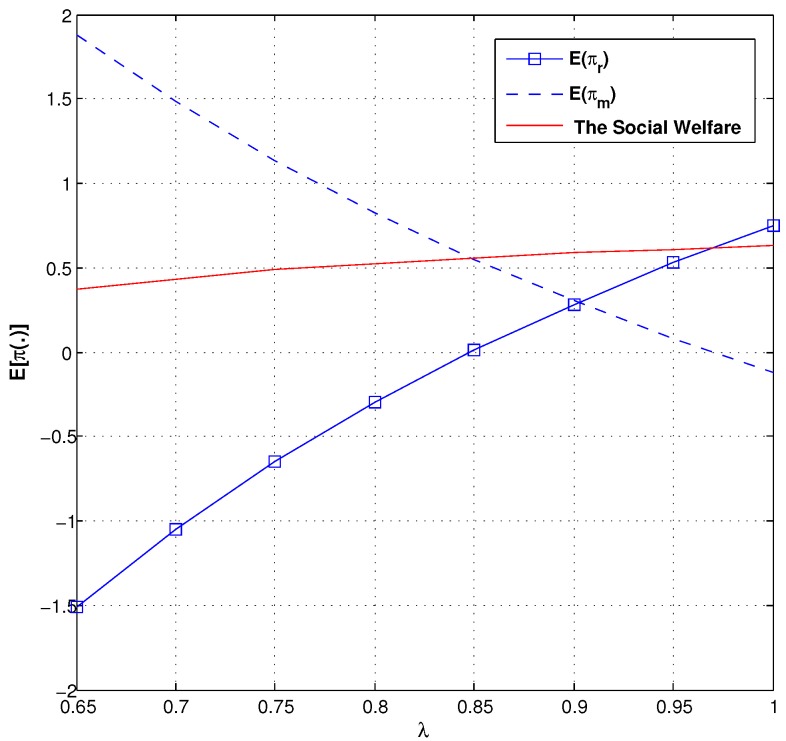
Description of social welfare and λ.

**Figure 9:**
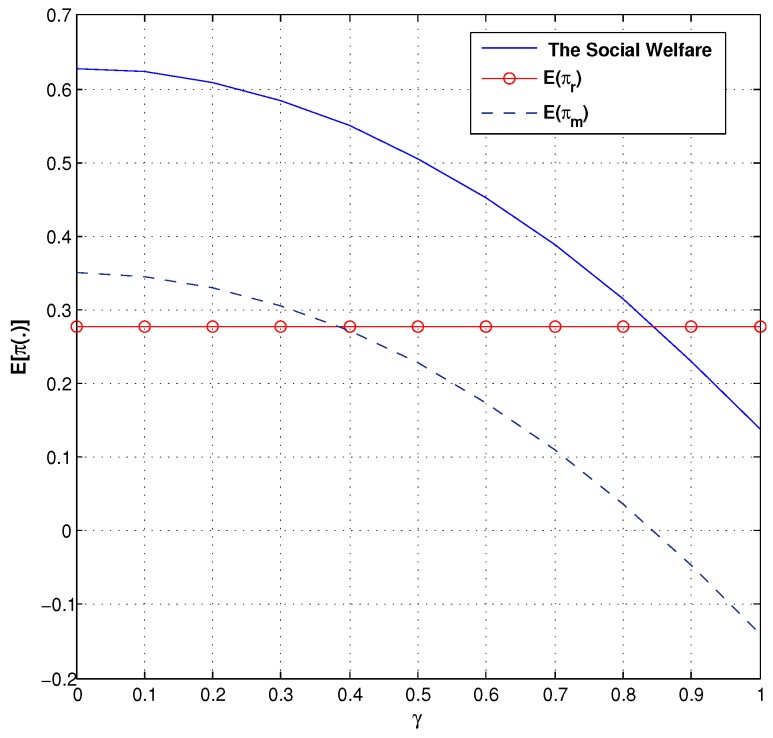
Description of social welfare and γ.

We now characterize the effect of the trust coefficient and the consumer aversion coefficient on social welfare in the green supply chain. Comparing the trust coefficient and social welfare by Figure 8, we find that the manufacturer’s expected profit strictly decreases with λ, the retailer’s expected profit strictly increases with λ, and social welfare weakly increases with λ. From Figure 9, we analyze the relationship between the consumer aversion coefficient and social welfare. We can see that both social welfare and the manufacturer’s expected profit strictly decrease with γ.

### 6.2. Environment Issue

**Figure 10:**
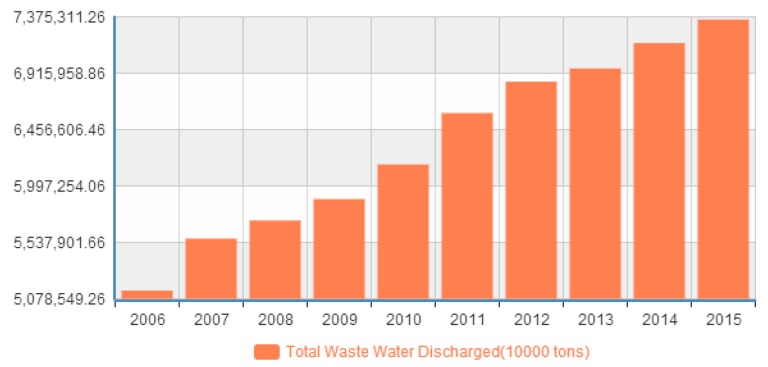
The total waste water discharged (1000 tons) in China.

**Figure 11:**
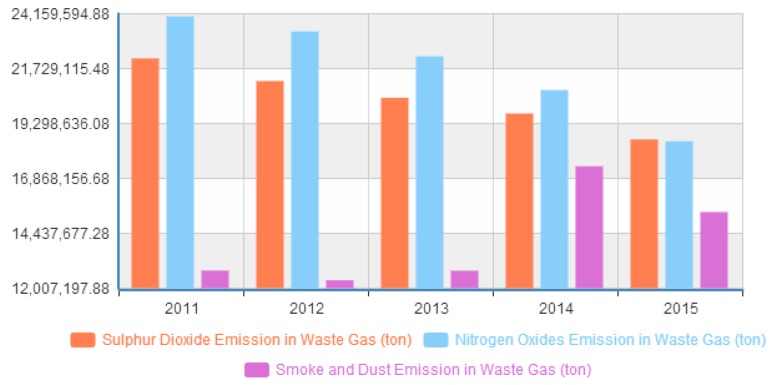
The waste gas discharged (1000 tons) in China.

This paper also uses 2006–2015 data of main pollutant emissions in waste water and gas by region in China, and shows Chinese environment is currently facing many serious challenges. In particular, Figure 10 shows the total waste water discharged in China between 2006 and 2015, and we can see that the total waste water emissions are increasing. In Figure 11, we have the main pollutant emissions in waste gases, including smoke and dust, sulphur dioxide, and nitrogen oxides in China in from 2011 to 2015. We can know that the exhaust gas emissions are weakly controlled by government policy and regulations.

**Figure 12:**
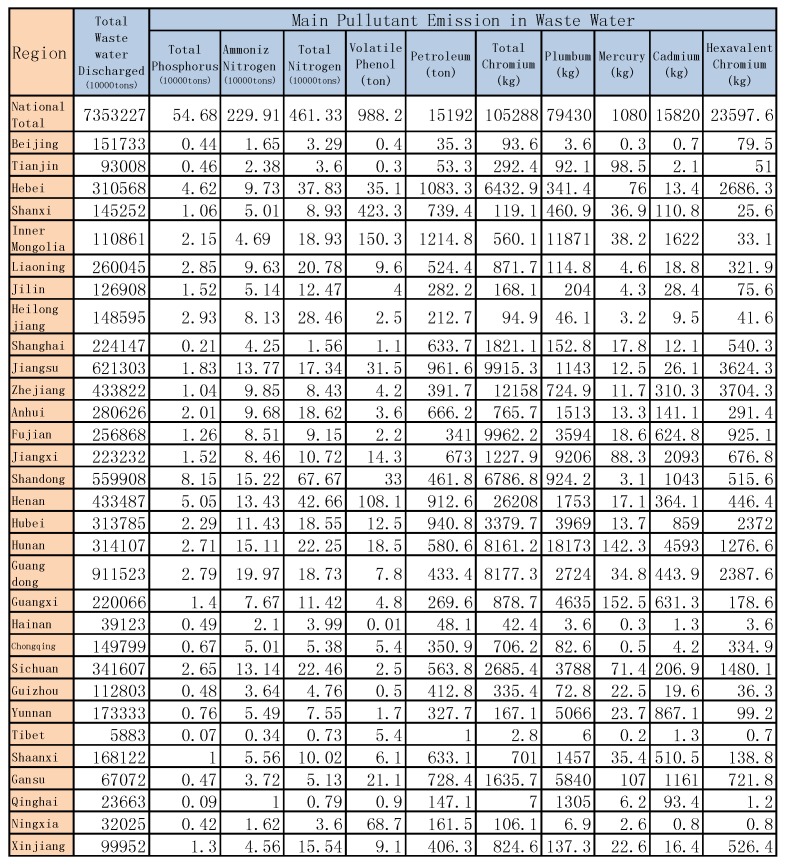
The main pollutant emissions in waste water by region in China in 2015.

**Figure 13:**
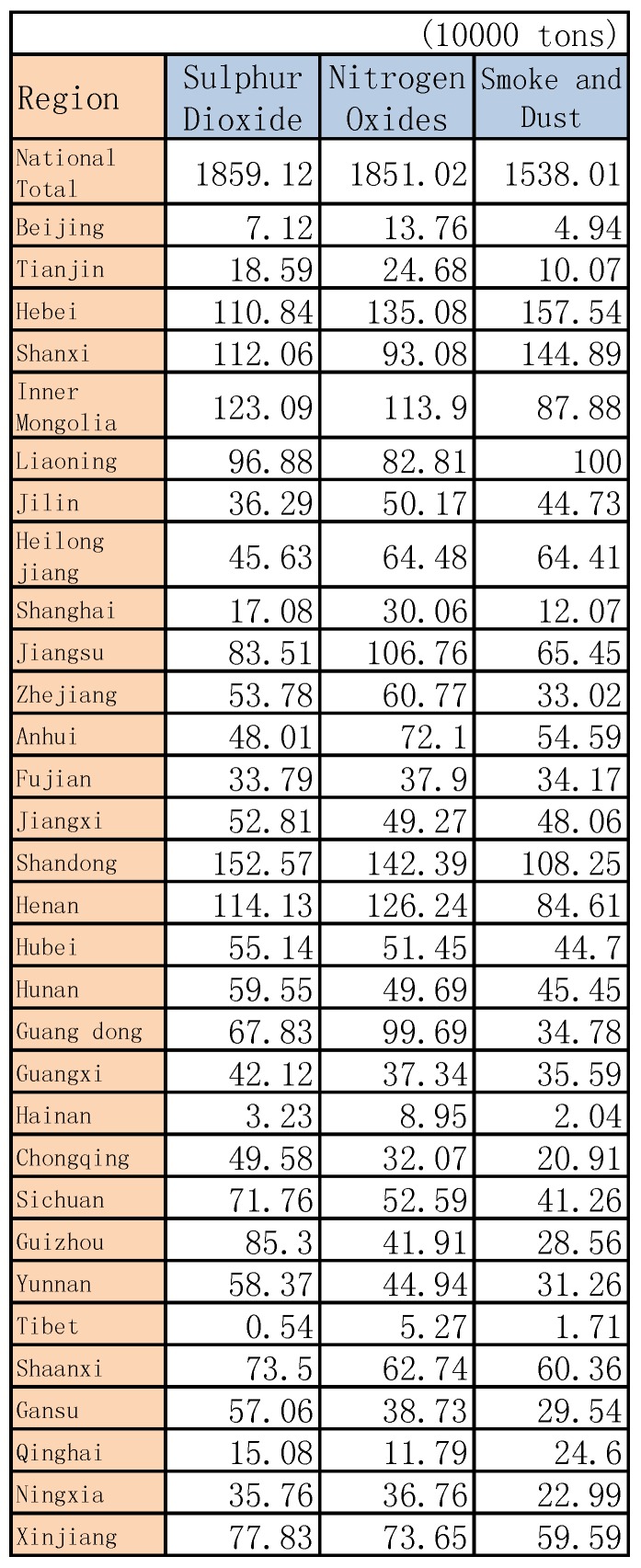
The main pollutant emissions in waste gases by region in China in 2015.

In addition, Figure 12 shows the main pollutant emission waste water by region in China in 2015, and Figure 13 shows the main pollutant emission waste gas by region in China in 2015; other years are similar (see Appendix B).

From the numerical examples, we come to the following conclusions:

(1) The numerical examples shows the green supply chain can operate optimally by considering information sharing, the consumer aversion coefficient, the trust coefficient, and rebate. In addition, our numerical examples offer the main properties of the game with a newsvendor model and drive a few managerial insights. Moreover, our work has proved that the method is correct and conclusions are reasonable in this paper.
(2) The data on emissions in China show the necessity for the government environment laws and regulations, and firms should produce more low-carbon products.

## 7. Conclusions

In this paper, we studied uncertain demand that has an impact on new low-carbon products by using a newsvendor model, and then we examined a game in which one manufacturer produces and delivers a single new low-carbon product to a single retailer. The retailer observes the demand information and gives an order before the selling season. Based on the previous studies, our work focused on the green supply chain which achieves coordination by considering subsidies, pricing, information sharing, the consumer aversion coefficient, trust coefficient, rebate, and other factors. Through the numerical examples we have shown that our conclusions are reasonable in this paper. Moreover, we used 2006–2015 data on the main pollutant emissions in waste water and gas by region in China to show Chinese environmental problems. Furthermore, the model we have studied in this paper tries to address these issues and provides new insights into low-carbon products, the supply chain coordination, information sharing, and order policy, enriching the previous studies. Through the above discussions, the major managerial insights of this paper are summarized as follows.

Firstly, the retailer shares truthful information, and the manufacturer then gives a low wholesale price at the beginning of selling season. However, when the retailer does not share or even shares unreal information, the manufacturer bears some risks, so he will set a trust coefficient to entice the retailer to share more truthful information. We know the manufacturer expects to enlarge the coverage of the product in market in practice, while as a rational policy-maker the retailer also will order more when he gains a low wholesale price under large demand.

In addition, when other factors are neglected, the manufacturer’s lot size was larger in previous studies. In our paper, the manufacturer has a unique lot size to achieve his expected profit optimally by considering the consumer aversion coefficient. When the retailer’s optimal order quantity is smaller than the manufacturer’s optimal lot size, we also found that the manufacturer will encourage the retailer to share more truthful information, instead of enticing the retailer to order more.

Moreover, this paper took the trust coefficient and consumer aversion coefficient as two qualitative variables into our model, as well as finding that the retailer’s optimal order quantity increases with the trust coefficient, while the manufacturer’s optimal supply quantity decreases with the consumer aversion coefficient. Hence, our model suggests the retailer should share more truthful forecast information to obtain a low wholesale price; the manufacturer should set new contracts including the trust coefficient and rebate to stimulate the retailer to share more.

In addition, waste water and exhaust gas emissions are a great danger to our life in practice, so our work is very meaningful. On the one hand, the development of new low-carbon products improves the quality of the environment. On the other hand, in order to alleviate global warming, governments and organizations should create laws and regulations to prohibit carbon emissions from firms, and entice firms to produce low-carbon products through financial subsidies, which will encourage all consumers to practice low-carbon consumption and promote the development of the low-carbon economy.

Finally, in this paper, our conclusions enrich and develop the use of the newsvendor model in uncertain demand. Based on the previous literature, these conclusions are helpful for the supply chain to operate optimally. The analysis of these conclusions suggests that the members should share more truthful information with each other. There are several worthy topics for possible further research. Due to the complexity of the problem, we only examine a single manufacturer’s optimal supply quantity under consideration of the trust coefficient and the consumer aversion coefficient. The wholesale price could be a key variable in our model, as as could be the manufacturer setting a wholesale price contract. In addition, our model could have two manufacturers and a single retailer, or a single manufacturer and two retailers. We will study how the members coordinate a green supply chain in future work.

## Figures and Tables

**Table 1:** Notations.

Symbol	Explanation
*p*:	retail price per unit of the retailer
*c*:	low-carbon product production cost per unit
*w*:	wholesale price per item of the manufacturer
*s*:	low-carbon product salvage value per unit
*D*:	market final demand for the low-carbon product
$L_{1}$:	the retailer obtains the government’s subsidy
$L_{2}$:	the manufacturer obtains the government’s subsidy
F(.), f(.):	prior distribution and density function of *D*
$\pi_{m}\left(.\right)$:	manufacturer profit
$\pi_{r}\left(.\right)$:	retailer profit
$\pi_{s}\left(.\right)$:	supply chain profit

**Figure A1:**
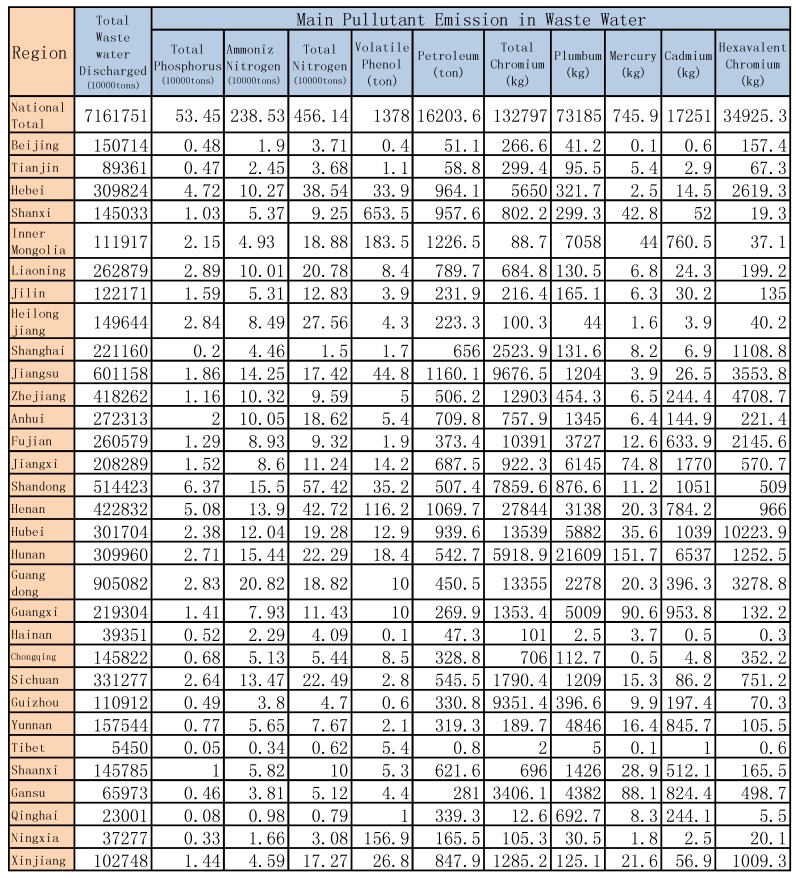
The main pollutant emissions in waste water by region in China in 2014.

**Figure A2:**
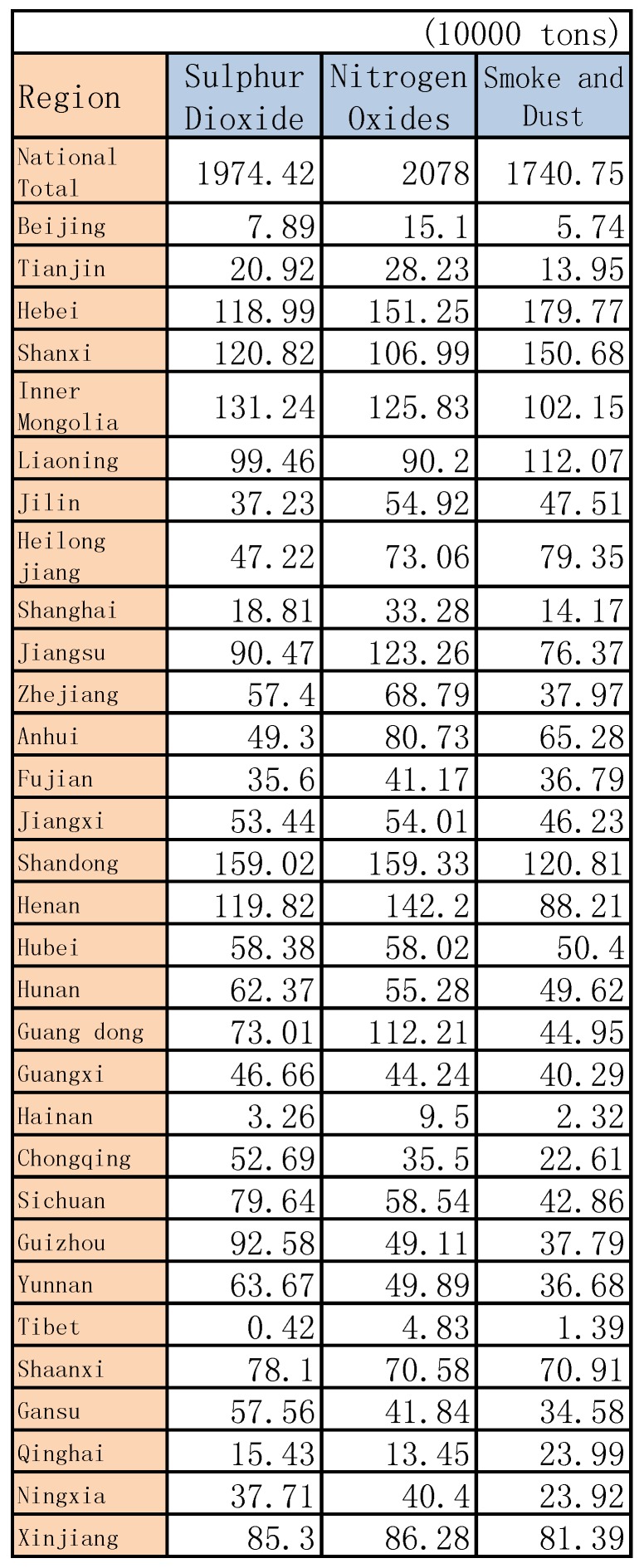
The main pollutant emissions in waste gas by region in China in 2014.

**Figure A3:**
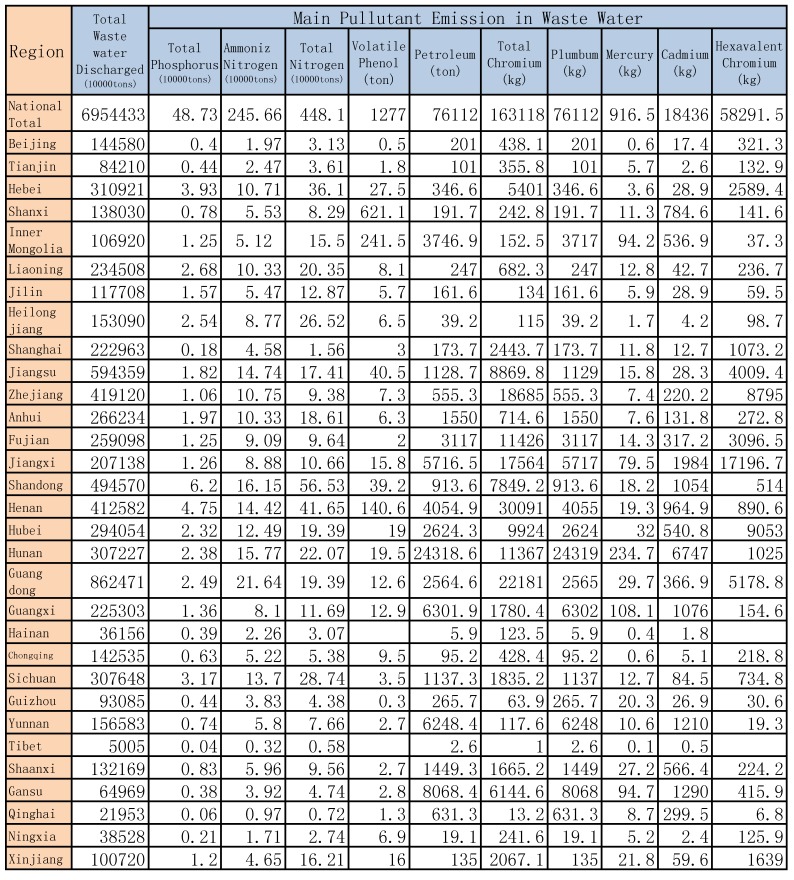
The main pollutant emissions in waste water by region in China in 2013.

**Figure A4:**
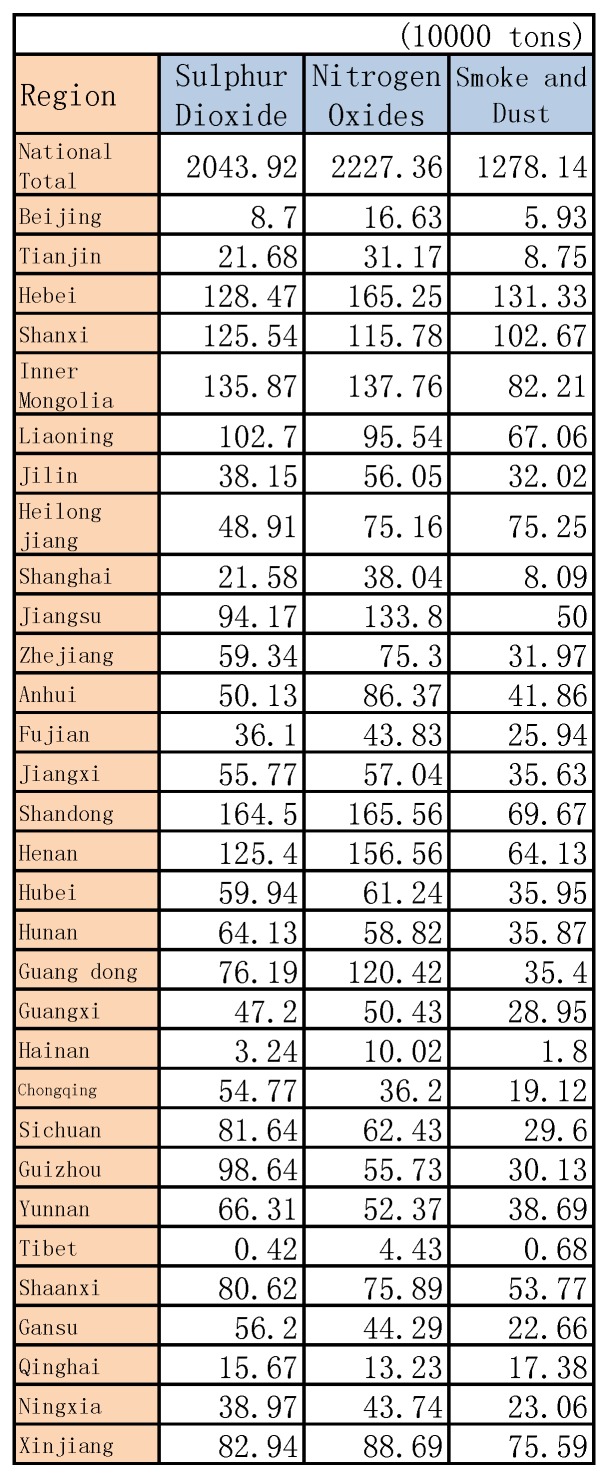
The main pollutant emissions in waste gas by region in China in 2013.

## Appendix A

**Proof of Proposition** **1.** In Equation (3), taking the first derivative of $q_{r}$, we have

(A1) $$\frac{\partial q_{r}}{\partial p}=f\left(F^{-1}\left(\frac{p-w+L_{1}}{p-s}\right)\right)\frac{w-s-L_{1}}{{\left(p-s\right)}^{2}}.$$

We can see by Equation (A1) that if $w\in (c,s+L_{1})$, $q_{r}$ is decreasing with *p* since $\frac{\partial q_{r}}{\partial p}<0$. When the demand is active, the manufacturer gives a low *w* to entice the retailer to increase his $q_{r}$. In this case, if the retailer posts a higher *p*, a small number of consumers will purchase the product since his $q_{r}$ is decreased. On the contrary, the majority of consumers are met. Therefore, we believe that the retailer as a rational policy maker can order more products. ☐

**Proof of Theorem** **1.** Let $A=\frac{(w-s)q}{p+L_{1}-s}$; we find the manufacturer’s expected profit:

(A2) $$\begin{array}{cc} E[\pi_{m}\left(\gamma ,q\right)] & =\int_{0}^{A}\left\{\gamma \left[\left(p+L_{1}\right)D+s\left(q-D\right)\right]+\left(1-\gamma \right)wq-cq+L_{2}q-c_{m}\right\}f\left(x\right)dx\\ & +\int_{A}^{\infty }\left[\left(w+L_{2}-c\right)q-c_{m}\right]f\left(x\right)dx. \end{array}$$

The simplified form of Equation (A2) is

(A3) $$E\left[\pi_{m}\left(\gamma ,q\right)\right]=\left(w+L_{2}-c\right)q-\int_{0}^{A}\left(p+L_{1}-s\right)\gamma F\left(x\right)-c_{m}dx.$$

Next, we take the first and second derivatives of $E[\pi_{m}\left(\gamma ,q\right)]$ with *q*, respectively. We have

(A4) $$\frac{\partial E[\pi_{m}\left(\gamma ,q\right)]}{\partial q}=w+L_{2}-c-\gamma \left(w-s\right)F\left(A\right),$$

(A5) $$\frac{\partial^{2}E\left[\pi_{m}\left(\gamma ,q\right)\right]}{\partial q^{2}}=\frac{-\gamma {\left(w-s\right)}^{2}f\left(A\right)}{p+L_{1}-s}.$$

As γ>0, $p+L_{1}-s>0$, f(.)>0, we can get $\frac{\partial^{2}E\left[\pi_{m}\left(\gamma ,q\right)\right]}{\partial q^{2}}<0$. Then, let $\frac{\partial E[\pi_{m}\left(\gamma ,q\right)]}{\partial q}=0$. We have $q_{m}=\frac{p+L_{1}-s}{w-s}F^{-1}\left(\frac{w+L_{2}-c}{\gamma (w-s)}\right)$. Hence, the optimal supply quantity is $q_{m}$. That is, the manufacturer has a unique maximizer of $E[\pi_{m}\left(\gamma ,q\right)]$ when $q_{m}=\frac{p+L_{1}-s}{w-s}F^{-1}\left(\frac{w+L_{2}-c}{\gamma (w-s)}\right)$. ☐

**Proof** **of** **Theorem** **2.** As shown in Equation (8), we can get the retailer’s optimal order quantity $q_{r}=F^{-1}\left(\frac{p-\frac{1}{\lambda }w+L_{1}}{p+L_{1}-s}\right)$. Next, taking the first derivative of $q_{r}$ on λ, we have

(A6) $$\frac{\partial q_{r}}{\partial \lambda }=f\left(F^{-1}\left(\frac{p-\frac{1}{\lambda }w+L_{1}}{p+L_{1}-s}\right)\right)\frac{w}{\left(p+L_{1}-s\right)\lambda^{2}}.$$

It is clear that $\frac{\partial q_{r}}{\partial \lambda }>0$. We find that $q_{r}$ is increasing with λ. In addition, when the demand is great, the retailer needs to order more products to meet the current market demand. Through the above discussions, we learn that if the retailer orders more, he will gain a good impression from the manufacturer, and at the same time, the manufacturer can give a high trust coefficient to his retailer. Hence, when the demand is large, the retailer prefers to share more truthful information with manufacturer in the new mechanism. This concludes the proof. ☐

**Proof** **of** **Theorem** **3.**

1. From the Equation (9), we have and simplify the manufacturer’s expected profit:
  (A7) $$\begin{array}{cc} E[\pi_{m}\left(\lambda ,\gamma ,q\right)] & =\int_{0}^{B}\left\{\gamma \left(p+L_{1}\right)D+s\left(q-D\right)+\left(1-\gamma \right)\frac{1}{\lambda }wq-cq+L_{2}q-c_{m}\right\}f\left(x\right)dx\\ & +\int_{B}^{\infty }\left[\left(\frac{1}{\lambda }w+L_{2}-c\right)q-c_{m}\right]f\left(x\right)dx\\ & =\left(\frac{1}{\lambda }w+L_{2}-c\right)q-\int_{0}^{B}\left(p+L_{1}-s\right)\gamma F\left(x\right)dx. \end{array}$$
  We can get obtain his optimal order quantity $q_{m}=\frac{p+L_{1}-s}{\frac{1}{\lambda }w-s}F^{-1}\left(\frac{\frac{1}{\lambda }w+L_{2}-c}{\gamma (\frac{1}{\lambda }w-s)}\right)$. Next, we take the first derivative of $q_{m}$ on λ, i.e.,
  (A8) $$\frac{\partial q_{m}}{\partial \lambda }=\frac{w(p+L_{1}-s)}{{\left(w-\lambda s\right)}^{2}}\left[F^{-1}\left(H\right)+\frac{\lambda (L_{2}-c+s)}{\gamma (w-\lambda s)}f\left(F^{-1}\left(H\right)\right)\right]$$
  For any $\gamma \in [\frac{\left(L_{2}-c+s\right)f\left(F^{-1}\left(H\right)\right)}{F^{-1}\left(H\right)s},1]$, when $\lambda >\frac{\gamma wF^{-1}\left(H\right)}{\gamma F^{-1}\left(H\right)s-\left(L_{2}-c+s\right)f\left(F^{-1}\left(H\right)\right)}$, we can get $\frac{\partial q_{m}}{\partial \lambda }<0$. As a result, the manufacturer’s optimal lot size is decreasing with the trust coefficient. It is easy to understand that when the consumer aversion coefficient is large, if the manufacturer posts a high trust coefficient, the manufacturer will bear more risks in the whole selling season. Hence, when the retailer obtains a high λ by sharing more information, the manufacturer will produce and deliver less than the retailer’s order quantity.
2. Similarly, we also have
  (A9) $$\begin{array}{cc} \frac{\partial q_{m}}{\partial \gamma } & =-\frac{p+L_{1}-s}{\frac{1}{\lambda }w-s}\frac{w+L_{2}-c}{\left(w-\lambda s\right)\gamma^{2}}f\left(F^{-1}\left(H\right)\right)\\ & =-\frac{\lambda \left(p+L_{1}-s\right)\left(w+L_{2}-c\right)}{{\left(w-\lambda s\right)}^{2}\gamma^{2}}f\left(F^{-1}\left(H\right)\right). \end{array}$$
  Since λ∈[0,1], p>s and w>c, we can get that $\frac{\partial q_{m}}{\partial \gamma }<0$. Hence, $q_{m}$ is decreasing with γ, that is, the manufacturer’s optimal lot size is decreasing with the consumer aversion coefficient.

 ☐

**Proof** **of** **Proposition** **2.**

1. Taking the first derivative of $E[\pi_{m}\left(\lambda ,\gamma ,b_{1},q\right)]$ with λ, we have $\frac{\partial E[\pi_{m}\left(\lambda ,b_{1},q\right)]}{\partial \lambda }=-\frac{wq}{\lambda^{2}}<0$. As a result, the manufacturer’s expected profit is decreasing with the trust coefficient when the demand is large enough under γ→0. In addition, it is obvious that the retailer’s expected profit is increasing with λ.
  The manufacturer makes the strategy decision that he gives a small λ when the demand is great enough. However, in order to maximize expected profit, the retailer is willing to share more truthful forecast information to increase his λ in the game. Further, we find that the manufacturer benefits from the decision since he can obtain more truthful demand information. Unfortunately, the supply chain is not coordinated. To settle the issue, the manufacturer offers a contract in which the retailer gains a rebate from the manufacturer after ordering the low-carbon product per unit. If $b>\Delta \frac{1}{\lambda^{\prime }}w$ ($\Delta \frac{1}{\lambda^{\prime }}=\frac{1}{\lambda^{c}}-\frac{1}{\lambda^{p}}$, $\lambda^{c}$ is the current trust coefficient, and $\lambda^{p}$ is the previous coefficient), the retailer will prefer to accept the manufacturer’s decision when the demand is large enough. Hence, we find that the retailer is likely to obtain a small λ.
2. In Equation (13), taking the first derivative of $E[\pi_{m}\left(\lambda ,\gamma ,b_{1},q\right)]$ with λ, we have
  (A10) $$\begin{array}{cc} \frac{\partial E[\pi_{m}\left(\lambda ,\gamma ,b_{1},q\right)]}{\partial \lambda } & =-\frac{wq}{\lambda^{2}}+\gamma F\left(c\right)-\frac{wq}{\lambda^{2}}\\ & =\frac{wq}{\lambda^{2}}\left[\gamma F\left(c\right)-1\right]. \end{array}$$
  As γ∈[0,1], F(.)≥0, and γ≤1, we have $\frac{\partial E[\pi_{m}\left(\lambda ,\gamma ,b_{1},q\right)]}{\partial \lambda }<0$. Hence, the manufacturer’s expected profit is decreasing with the trust coefficient when the demand is depressed by considering the consumers’ coefficient.
  In general, the retailer shares unreal (or even does not share) information to the manufacturer when the demand is depressed. However, in order to obtain optimal expected profit, the manufacturer will make a new contract setting a rebate $b_{1}$ in the game. We find that the retailer will prefer to share more truthful information if the $b_{1}$ is set. At the same time, the manufacturer can give a higher λ.

 ☐
